# Receiving an Alcohol Enquiry from a Physician in Routine Health Care in Sweden: A Population-Based Study of Gender Differences and Predictors

**DOI:** 10.3390/ijerph8051296

**Published:** 2011-04-27

**Authors:** Barbro Engdahl, Per Nilsen

**Affiliations:** 1 Centre for Social Research on Alcohol and Drugs, SoRAD, Stockholm University, SE-106 91 Stockholm, Sweden; 2 Department of Medical and Health Sciences, Linköping University, SE-581 83 Linköping, Sweden; E-Mail: per.nilsen@liu.se

**Keywords:** alcohol enquiry, health care, gender, hazardous drinking, alcohol-related problems, social class

## Abstract

Research has shown that the provision of brief interventions in the health care system is effective for reducing hazardous drinking. Using a telephone-administered questionnaire, this study provides a population-based investigation on the extent to which physicians address patients’ alcohol habits in the Swedish health care system, whether there are gender differences in the extent to which patients receive questions about alcohol, and predictors for receiving such questions. Data were obtained from monthly telephone surveys with around 72,000 people in 2006–2009. Having received an alcohol enquiry was defined as having been asked about one’s drinking habits by a physician in any health care visit in the last 12 months. Fourteen percent of the total population had received an alcohol enquiry, but there were considerable gender differences: for hazardous drinkers, 13% of the women and 17% of the men had received an alcohol enquiry; among those with sensible alcohol consumption, 10% of women and 15% of men had received an alcohol enquiry. Patients were more likely to have received an alcohol enquiry if they had self-reported alcohol-related problems, were hazardous drinkers and/or daily smokers. Some of the alcohol enquiry predictors differed by gender; social class was an important predictor for women but not for men.

## 1. Introduction

Alcohol prevention has become a public health strategy in the last few decades. It has been recognized that most alcohol-related harm on a population level is attributable to the large group of drinkers who drink above the guidelines usually recommended by medical or government authorities, rather than individuals with severe alcohol-related problems or alcohol dependence. The result has been a shift in attention from treating solely dependent drinkers at one end of the continuum to secondary preventive interventions targeting individuals at risk of alcohol-related harm [1].

The shift towards a broader view of the alcohol problem has led to an increased expectation for health care providers to become more actively involved in secondary prevention. Brief intervention (BI) emerged in the 1980s as a strategy for use in general health care settings to provide early intervention, before or soon after the onset of alcohol-related problems for non-dependent patients. BIs are opportunistic interventions intended for a broad range of patients who utilize health care services but do not seek treatment related to their alcohol consumption [2]. The primary target groups for BI are those who drink above recommended guidelines: hazardous drinkers who are at physical, mental or social risk from alcohol consumption and harmful drinkers who are already experiencing physical, mental or social harm due to their consumption [3]. BIs can range from simply inquiring about a patient’s alcohol consumption to providing multiple sessions of counseling. The aim is to moderate drinking rather than attain complete abstinence [4,5].

Despite a solid evidence base that supports the efficacy of BI at reducing hazardous and harmful alcohol consumption, implementation of BI in various health care settings appears to have been slow [6]. However, the extent to which alcohol is actually addressed in routine health care has been sparsely researched. Exit poll surveys have been undertaken to investigate the extent to which alcohol is addressed in routine health care; respondents in these studies provide data immediately after their visits to health care providers. However, such studies focus on local clinical populations and do not provide information about the extent to which alcohol is addressed in the general public.

Previous alcohol research has pointed to gender differences in the extent to which patients receive alcohol interventions or treatment in various health care settings. In general, it has been found that men are more likely than women to discuss their alcohol consumption and/or receive treatment for alcohol-related problems in health care [7–11]. The higher likelihood of addressing male alcohol consumption is usually attributed to the higher prevalence of alcohol-related problems among men. It has also been argued that heavier drinking is considered a more deviant behavior for women than for men [12,13], which means that sociocultural norms and the social stigma related to drinking may reduce the likelihood that women seek help or are willing to discuss their alcohol problems with health care providers [14]. However, most of this research has focused on patients with more severe alcohol problems or dependence. Potential gender differences with regard to the addressing alcohol with non-dependent, non-treatment-seeking patients in routine health care have not been investigated.

This study attempts to fill critical knowledge gaps concerning alcohol prevention in health care for the broader public. Using a telephone-administered questionnaire, the study provides a population-based investigation into the extent to which physicians address patients’ alcohol habits in the Swedish health care system, whether there are gender differences in the extent to which patients receive questions about alcohol, and predictors for receiving an alcohol enquiry.

## 2. Methods

### 2.1. Study Design, Population and Data Collection

The study was based on a cross-sectional sample of 72,079 people, representative of the Swedish-speaking population aged 16–80 years with respect to age, gender and indicators of socioeconomic status such as educational level. Data were collected during the period January 2006 to December 2009.

The data were collected as part of the so-called Monitoring Project at the Centre for Social Research on Alcohol and Drugs (SoRAD) at Stockholm University [15]. The Monitoring Project is a monthly telephone-administered survey questionnaire with 1,500 respondents, using computer-aided telephone interviews. The Monitoring Project has been responsible for assembling data for official alcohol statistics in Sweden since 2000. Thirty contact attempts are made before a contact attempt is recorded as a non-response.

### 2.2. Study Variables

Sociodemographic data were obtained using four variables: gender, age, education level, and occupational status. Four age categories were constructed: 16–29 years; 30–49 years; 50–64 years; and 65–80 years. Education was categorized into: compulsory; upper secondary; and university. Occupational status was categorized into: employed or self-employed; student; unemployed, sick-listed or early retirement; retired pensioner.

Health care utilization was examined with a question about whether the respondent had visited a physician in the past 12 months. Respondents who answered “yes” to this question were then asked “Did your physician ask you about your alcohol habits?” An affirmative answer to this question is defined as having received an alcohol enquiry in this study.

Smoking was examined with a question on whether the respondent smoked daily in the last 30 days (measured from January 2006 to August 2009). Alcohol problems were investigated using 6 items: whether the respondents in the past 12 months had: (1) experienced a hangover; (2) been involved in an alcohol-related quarrel; (3) been involved in an alcohol-related fight; (3) sustained an alcohol-related accident; (5) experienced economic problems due to alcohol consumption; and (6) been a passenger in a car with a drunk driver.

Data on the respondents’ alcohol consumption was obtained from questions about the frequency of drinking and typical quantity of drinking in the last 30 days. Two categories of alcohol consumers were constructed on the basis of the answers on the two drinking variables: sensible drinkers and hazardous drinkers. Hazardous drinkers had a weekly consumption of >9 drinks for women (>15 g/day) and >14 drinks for men (>24 g/day), in accordance with the threshold for hazardous weekly alcohol consumption proposed by the Swedish National Institute of Public Health [16]. One Swedish drink equals 12 g of pure alcohol. Abstainers in the last 30 days and respondents drinking below this threshold were categorized as sensible drinkers.

### 2.3. Data Analysis

Data were analyzed using the statistical software package SAS 9.1 for Windows. Statistical analysis initially took the form of descriptive statistics plus a crude analysis of relationships between variables, χ^2^-tests for categorical data. The data were weighted with regard to municipal population size and age. The second phase of the analysis involved the development of a logistic regression model, based on crude analysis and theoretic criteria about independent variables that may influence the receipt of an alcohol enquiry. Statistical significance was analysed with the Wald test and was accepted at *p* < 0.05. Odds ratio (OR) plus 95% confidence intervals (95% CI) were calculated in the logistic regression.

The first and second analyses compared women and men with sensible and hazardous alcohol consumption measured by percentages. In the second descriptive analysis, the gender differences were examined as percentages in each category of the background variables: age, education level, occupational status, alcohol-related problems and smoking.

The third analysis was a logistic model with the outcome likelihood of having received an alcohol enquiry when visiting a physician in the last 12 months, separate for women and men in 4 models. The first regression model only included the level of alcohol consumption and smoking; the second model also included alcohol-related problems and controlled for age, which seems to be an important factor when studying health care utilization. The third model also included education level and occupational status. The values were weighted for age, sex and size at the municipality level in all the analyses.

## 3. Results

The average response rate was 45% in the monthly surveys during the study period. Approximately 43% of the study sample had used health care services in the previous 12 months. Women visited health care to a greater extent than men (58% *versus* 42%). The proportion of hazardous drinkers was 9% among the women and 14% among the men.

Table 1 shows the extent to which the female and male hazardous and sensible drinkers were asked about their alcohol habits in the previous 12 months. Overall, 14% of the total population received an alcohol enquiry. There were considerable gender differences. More men than women received an alcohol enquiry; this applied to both sensible and hazardous drinkers. Thirteen percent of the female hazardous drinkers received an alcohol enquiry compared with 17% of the male hazardous drinkers. Fifteen percent of the male sensible drinkers received an alcohol enquiry, more than the proportion of female hazardous drinkers (13%). Hazardous drinkers reported receiving an alcohol enquiry to a greater extent (15%) than the sensible drinkers (12%).

Table 2 provides data on the male and female hazardous and sensible drinkers who received an alcohol enquiry (sociodemographic characteristics, how many were smokers and how many reported alcohol-related problems). In all age groups, more men received an alcohol enquiry than women in the corresponding age groups, with the exception of male hazardous drinkers aged 16–29 years who did not receive an alcohol enquiry more often than women of the same age at a statistically significant level (*p* < 0.05). The gender differences concerning age applied to both sensible and hazardous drinkers.

Regardless of education level, men received an alcohol enquiry more than women with the same level of education. An exception was men with compulsory education who were hazardous drinkers, who did not receive alcohol enquiries more often than female hazardous drinkers with the same level of education.

More employed and unemployed men received an alcohol enquiry than women with the same occupational status. This applied to both sensible and hazardous drinkers. There were no gender differences with regard to alcohol enquiries received by students. Hazardous drinkers who were pensioners also did not differ in gender, but among sensible drinkers who were pensioners more men received an alcohol enquiry than women.

Among people who smoked, more men received an alcohol enquiry than women; this applied to both sensible and hazardous drinkers. A larger proportion of men who reported 0–2 alcohol-related problems received an alcohol enquiry than women with the same amount of problems. This result was valid for both hazardous and sensible drinkers. The gender differences disappeared when the patients had 3 or more alcohol-related problems. Among hazardous drinkers with 5 or 6 alcohol-related problems, more women were asked about alcohol consumption than men although this difference was not statistically significant.

Table 3 presents the odds ratios of having received an alcohol enquiry. Model 2 controlled for age and alcohol-related problems and model 3 also controlled for education level and occupational status. Gender differences were small but statistically significant when analyzing alcohol enquiries in relation to patients’ level of alcohol consumption (hazardous or sensible drinking). Female hazardous drinkers were 1.25 times more likely than female sensible drinkers and male hazardous drinkers 1.21 times more likely than male sensible drinkers to receive an alcohol enquiry. These odds ratios were lower in models 2 and 3, but male hazardous drinkers were 1.15 times more likely than male sensible drinkers to receive an alcohol enquiry after controlling for all the factors in model 3.

Female smokers were 1.32 times and male smokers were 1.36 times more likely than non-smoking women and men, respectively, to have received an alcohol enquiry. The odds ratios were lower in models 2 and 3, but remained statistically significant.

Women who reported 3–4 alcohol-related problems were 1.6 times more likely and those who had experienced 5–6 problems were 3.4 times more likely than women who reported 0–2 problems to have received an alcohol enquiry. The difference in likelihood of having received an alcohol enquiry was smaller for men. However, men who reported 5–6 problems were 2 times more likely than men reporting 0–2 problems to have received an alcohol enquiry. The odds ratios were lower after controlling for the factors in model 3, but remained significant.

Younger women were more likely to have received an alcohol enquiry; those aged 16–29 years were two times more likely than women aged 65 years or more to have received an alcohol enquiry. However, the effect of age largely disappeared when controlling for education level and occupational status in model 3. However, men aged 30–64 years were 1.3–1.4 times more likely than men aged 65 years or more to have received an alcohol enquiry.

Women who were unemployed, sick-listed or early retired were 1.6 times more likely and female pensioners were less likely to have received an alcohol enquiry than employed women. There was no statistically significant effect of occupational status on the likelihood of men receiving an alcohol enquiry.

## 4. Discussion

This study sought to investigate the extent to which physicians address patients’ alcohol habits in the Swedish health care system, whether there are gender differences in the extent to which patients receive an alcohol enquiry, and predictors for receiving an alcohol enquiry. Approximately 1 in 8 (12%) of those who had visited a physician in health care in the previous 12 months had discussed their alcohol consumption habits. There are few comparisons for our alcohol enquiry prevalence rate. The only other comparable data in a general population survey of which we are aware is the British National Statistics Opinion Survey [17], which is repeated every year or two. In 2009, 10% of the men and 7% of the women aged 16 years or more reported discussing their drinking with a health professional in the previous year. These rates have been largely unchanged since 2000 [17]. Our study only included consultations with physicians, whereas the British surveys cover all professional categories in health care, making comparisons difficult.

The prevalence of alcohol conversations in health care has also been investigated in exit poll surveys, which assess the extent to which patients leaving a health care center have been asked about their alcohol consumption during the visit to a health care provider. For instance, Aalto and Seppä [18] reported that 12% of Finnish primary health care patients in Tampere had been asked and/or advised about drinking alcohol during the consultation. Another exit poll study (modeled on the Finnish study) conducted at an outpatient clinic for students in St. Petersburg, Russia, found that 8% of the patients had been counseled on their alcohol consumption [19]. Differences in study designs and populations should be borne in mind when interpreting exit poll data in comparison with our results.

We found that receiving an alcohol enquiry was more likely when the respondents reported having alcohol-related problems. This applied to both women and men, but there was a tendency for more women with several alcohol-related problems to receive an alcohol enquiry than men. People who have alcohol-related problems are more likely to have alcohol-related symptoms than people without these problems, and should thus be more easily identified to receive an alcohol enquiry.

Hazardous drinkers, men and women, received an alcohol enquiry more often than sensible drinkers. However, applying a certain consumption limit as a threshold to identify hazardous drinking, as we did in this study, means that there are also alcohol-dependent drinkers among this group of drinkers. This may provide a partial explanation for why the physicians addressed alcohol more often with hazardous drinkers than with sensible drinkers.

Daily smoking in the last 30 days also predicted receipt of an alcohol enquiry for women and men. Smoking is related to higher alcohol consumption [20]. A daily smoker is easily detected and knowledge of the smoking–drinking association probably increases the likelihood that physicians and other health care providers address alcohol with these patients.

Some of the predicting factors to receive an alcohol enquiry differed by gender. Occupational status was an important predictor for alcohol enquiry among women. More women who were unemployed, sick-listed or early retired received an alcohol enquiry than women who were working. Age seemed to be less important than occupational status for women. The unemployed are younger than the average population [21]; sick-listed and early retired persons are often older than the average in society [22]. Occupational status was not an important predictor for men’s receipt of an alcohol enquiry. Age and hazardous drinking were significant alcohol enquiry predictors for men. These results imply that the reasons for addressing alcohol with women are more complex than those for addressing alcohol with men.

There were considerable gender differences with regard to the receipt of alcohol enquiry; 15% of the men and 10% of the women reported having had their alcohol consumption addressed by a physician. There may be several explanations for this finding. An obvious reason is the higher prevalence of alcohol-related problems among men [15], which probably influences physicians to address alcohol issues more often with men than women. Physicians likely have more experience in handling male alcohol problems and may feel more comfortable than with female alcohol problems. Fifty-five percent of the physicians in Sweden are male [23], which may also explain why male patients received alcohol enquires more often than females. Gender differences in the medical conditions that men and women present with may also be a factor. It has been suggested that women’s alcohol-related problems have different signs or symptoms than men’s, in the same way as diseases such as cardiovascular disease have different signs for women than for men [24].

Other explanations for the gender differences should be considered. Public perception and attitudes on male and female alcohol consumption likely influence physicians’ decisions regarding the categories of patients they believe are most in need of alcohol preventive interventions. Women’s drinking has often been described as a symptom of insecurity, low self-esteem or a result of being in a state of dependence, with alcohol functioning as a form of self-medication to escape from negative elements in life. There are official Swedish policy documents that reinforce this view of female alcohol consumption, describing women’s drinking as a consequence of complex underlying problems of an individual and psychological nature [25]. In contrast, men’s drinking is typically viewed as less complex and easier to treat because their drinking is better tolerated by society. Men’s alcohol-related problems tend to be viewed as consequences of their alcohol consumption rather than being the cause of their drinking [26].

This study has some methodological shortcomings that must be considered when interpreting the results. We used self-reports to measure alcohol intake. Although alcohol self-reporting measures tend to be valid and reliable under most circumstances [27], some underreporting cannot be ruled out. The survey questionnaire did not include any questions about the content or duration of the alcohol discussions or whether the patients were screened for their alcohol consumption. Reported outcomes, both in relation to alcohol consumption and whether alcohol was addressed, are vulnerable to recall bias as well as underreporting for social desirability reasons. Our response rate of 45% is similar to other population-based health telephone surveys.

## 5. Conclusions

Approximately 1 in 8 of those who visited a physician in Swedish health care in the previous 12 months received an alcohol enquiry, *i.e.*, a physician addressed the patient’s alcohol habits. There were considerable gender differences with regard to receiving an alcohol enquiry; 10% of the women and 15% of the men in the study population received an alcohol enquiry. Patients were more likely to receive an alcohol enquiry if they had alcohol-related problems, were hazardous drinkers and/or daily smokers. Some of the alcohol enquiry predictors differed by gender; social class was an important predictor for women but not for men.

## Figures and Tables

**Table 1.** Proportion of women and men who visited a physician in health care and received an alcohol enquiry in the last 12 months, % and χ^2^-test.

	**Women (*N* = 38,821) *n***	**%**	**Men (*N* = 33,232) *n***	**%**	**χ^2^-value**	**p-value**
Visited a physician in the last 12 months	18,251	55	13,095	45	523.1	<0.0001
Received an alcohol enquiry						
Hazardous drinkers (*n* = 3,505)	1,630	13	1,875	17	15.6	<0.0001
Sensible drinkers (*n* = 27,841)	16,621	10	11,220	15	121.1	<0.0001

χ^2^–test between categories of women and men	Women		Men		Women and men	
Hazardous and Sensible drinkers	*(n* = 18,251)		*(n* = 13,095)			
χ^2^-value	9.8		10.0			
*p*-value	0.002		0.002			

Female Hazardous drinkers and Male Sensible drinkers	(*n* = 1,630)		(*n* = 11,220)		(*n* = 12,850)	
χ^2^-value					4.6	
*p*-value					0.03	

**Table 2.** Proportions of alcohol enquiries for hazardous and sensible drinkers by sociodemographic characteristics, smoking, and alcohol-related problems, % of each category and χ^2^–test between women and men.

	**Hazardous drinkers (*n* = 3,505)**			**Sensible drinkers (*n* = 27,741)**			**All (*n* = 31,246)**		

	**Women (*n* = 1,630) %**	**Men (*n* = 1,875) %**	**Sign**	**Women (*n* = 16,621) %**	**Men (*n* = 11,120) %**	**Sign**	**Women (*n* = 18,251) %**	**Men (*n* = 12,995) %**	**Sign**
*Educational level*									
Compulsory	16	20		10	13	***	10	14	***
Upper secondary	13	18	*	10	16	***	11	16	***
University	11	16	***	10	14	***	10	14	***

*Occupational status*									
Employed	11	17	***	10	16	***	11	16	***
Student	16	15		12	14		13	14	
Unemployed, sick-listed, early retired	15	24	*	14	17	***	14	18	**
Pensioner	9	14		6	10	*	6	11	***
Daily smoking	19	25	***	12	18	***	13	19	***

*Number of alcohol-related problems*									
0	9	16	***	9	14	***	9	14	***
1–2	13	19	**	11	15	***	11	16	***
3–4	21	18		18	19		19	18	
5–6	56	31		0	27		34	29	

*Age*									
16–29 years	14	16		13	15	***	14	15	*
30–49 years	12	19	***	12	16	***	12	17	***
50–64 years	14	19	*	10	16	***	11	17	***
65–80 years	7	13	*	6	10	***	7	12	***

χ^2^–test between women and men:

*** *p* < 0.001,

** *p* < 0.01,

* *p* < 0.05.

**Table 3.** Logistic regression of gender-specific odds ratios of having received an alcohol enquiry from a physician in health care in the last 12 months (*n* = 16,516 women, *n* = 11,762 men); 95 % CI and Wald test.

	**Model 1**		**Model 2**		**Model 3**			

	**Women**	**Men**	**Women**	**Men**	**Women**	**Wald**	**Men**	**Wald**
Hazardous alcohol consumption (with reference to sensible alcohol consumption)	1.25 **	1.21 **	1.13 *	1.15 *	1.15 (0.98–1.36)		1.15 (1.01–1.49)	*
Daily smoking	1.32 ***	1.36 ***	1.27 **	1.30 ***	1.23 (1.06–1.42)	*	1.28 (1.09–1.49)	**

Alcohol-related problems (with reference to 0–2 alcohol-related problems)								

0–2 problems			1	1	1		1	
3–4 problems			1.58 *	1.16	1.55 (1.05–2.26)	*	1.14 (0.82–1.58)	
5–6 problems			3.58 **	2.13 *	3.42 (1.56–7.51)	***	2.03 (1.08–3.76)	*

Educational level (with reference to compulsory level)								

Compulsory					1		1	
Secondary					0.91 (0.79–1.05)		1.06 (0.93–1.21)	
University					0.94 (0.8–1.09)		0.95 (0.82–1.1)	

Occupational status (with reference to employed)								

Employed					1		1	
Student					1.12 (0.91–1.38)		0.91 (0.71–1.16)	
Unemployed/sick-listed/early retired					1.55 (1.31–1.83)	***	1.13 (0.96–1.34)	
Pensioner					0.78 (0.63–0.97)	*	0.82 (0.63–1.08)	

Age (with reference to 65+ years)								

16–29 years			1.99 ***	1.42 ***	1.32 (0.9–1.93)		1.21 (0.88–1.65)	
50–64 years			1.59 ***	1.64 ***	1.08 (0.76–1.54)		1.36 (1.03–1.78)	*
65 years +			1	1	1		1	

Wald test was used to measure significance in the variables:

*** *p*<0.001,

** *p*<0.01,

* *p*<0.05.
